# Sexual and Reproductive Health: How Can Situational Judgment Tests Help Assess the Norm and Identify Target Groups? A Field Study in Sierra Leone

**DOI:** 10.3389/fpsyg.2022.866551

**Published:** 2022-05-05

**Authors:** Lisa Selma Moussaoui, Erin Law, Nancy Claxton, Sofia Itämäki, Ahmada Siogope, Hannele Virtanen, Olivier Desrichard, Consortium Sierra Leone Red Cross Society

**Affiliations:** ^1^Research Group in Health Psychology, Faculty of Psychology and Education, Université de Genève, Geneva, Switzerland; ^2^Finnish Red Cross, Helsinki, Finland; ^3^Nadulpan, Crestview, FL, United States; ^4^Sierra Leone Red Cross, Freetown, Sierra Leone

**Keywords:** gender-based violence, family-planning, child early and forced marriage, female genital mutilation and cutting, social norms, low- and middle-income countries, community assessment, situational judgment tests

## Abstract

Sexual and reproductive health is a challenge worldwide, and much progress is needed to reach the relevant UN Sustainable Development Goals. This paper presents cross-sectional data collected in Sierra Leone on sexual and gender-based violence (SGBV), family planning (FP), child, early and forced marriage (CEFM), and female genital mutilation (FGM) using an innovative method of measurement: situational judgment tests (SJTs), as a subset of questions within a larger survey tool. For the SJTs, respondents saw hypothetical scenarios on these themes and had to indicate how they would react. The objective is to give an impression of beliefs and norms on specific behaviors, which provide insights for social and behavior change interventions. Data was collected by enumerators traveling to villages randomly selected in six districts of the country. The sample is composed of 566 respondents. Results show that FGM in particular seem to be a priority topic, in comparison to the other topics for which the norms seem to be stronger against those practices. Age differences emerged and suggest priority groups to be targeted (e.g., on the topic of female genital mutilation, younger female respondents, and older male respondents gave the lowest coded responses which reflected to less appropriate behavior in our coding). In terms of validity of the measurement methods, situational judgment test answers correlated positively with other items in the survey, but the magnitude of the association is often small, and sometimes not significant. Thus, more studies are needed to further explore the validity of this measure by comparing against a reference value. Using SJTs could complement other data collection tools to perform community assessment, and orient the direction of the program in its planning phase.

## Introduction

Sexual and reproductive health is a challenge worldwide and is a foundation of many of the UN Sustainable Development Goals. While the target 3.7 of Goal 3 Good Health and Well-being is “By 2030, ensure universal access to sexual and reproductive healthcare services, including for family planning […],” the proportion of women of reproductive age who have their family planning (FP) needs met is around 77 percent globally, and only 56 percent in sub-Saharan Africa in 2021 (United Nations, 2021). A similar discrepancy exists between the current situation and the targets 5.3 of Goal 5 Gender Equality, “Eliminate all harmful practices, such as child, early and forced marriage, and female genital mutilation (FGM),” and target 5.2 “Eliminate all forms of violence against all women and girls in the public and private spheres […].” The document “Progress toward the Sustainable Development Goals” (United Nations, 2021) reports a dramatic situation. Estimates for the period 2000–2018 show that one in three women have been subjected to physical violence by an intimate partner, sexual violence, or both at least once in their lifetime. Child marriage declined by 15% from 2010 to 2020 but is expected to increase in the coming years due to the COVID-19 pandemic. FGM is still occurring in 31 countries. Nine out of 10 girls and women from 15 to 49 years have been mutilated in some places. Data from the Demographic and Health Survey 2019 in Sierra Leone show that those issues are pressing in this country: half of women age 25–49 gave birth for the first time before 20 years old (median = 19.5 years), and the proportion of current teenage childbearing is 21% [Statistics Sierra Leone (Stats SL) and ICF, 2020]. In terms of FP, 46% of married women having a demand (spacing births or limiting births), and more than half of those consider their needs unmet. According to the same survey, the percentage of women having experienced physical violence is 61%, and among ever-married women, the perpetrator of violence was often the partner. Similarly for sexual violence, experienced by 7% of the respondents, the most common perpetrator is the current or former partner. Female genital cutting concerns 83% of women between 15 and 49, which were mostly circumcised before 15 years old. Thus, more needs to be done in the field of sexual and reproductive health in Sierra Leone. The question is, how can we effectively tackle the challenges related to sexual and reproductive health?

Social and behavior change interventions are a way to modify the behavior behind the challenges mentioned above (World Health Organization, 2011; Collumbien et al., 2012; Brown et al., 2013; Spring et al., 2016; Cislaghi et al., 2019). In order to effectively change behavior, interventions need to target the correct drivers of behavior. For example, Huber et al. (2014) demonstrated that a tailored intervention targeting factors previously identified *via* a survey in the population was more effective to promote the uptake of the behavior (consumption of fluoride-free water in rural Ethiopia) compared to a traditional information intervention because in the target group the barriers to behavior were perceived costs and not a lack of information on the problem. Thus, data on what the target group believes and what the norms are is crucial to building effective social and behavior change interventions instead of relying on intuition (Wilson and Juarez, 2015), and to allow interventions to go further than only supplying information and education to raise awareness (World Health Organization, 2011).

Specifically, evidence-based effective interventions seem to be missing on the sexual and reproductive health topics targeted in this paper. For example, Berg and Denison (2012) conducted a systematic review on interventions to reduce the occurrence of FGM or “cutting” as referenced in fieldwork and concluded that interventions could have positive effects on attitudes, but not on the practice of FGM. In addition, a review by Lee-Rife et al. (2012) highlights that information is missing on the mechanism through which prevention programs carried out in low-income countries impact (or fail to impact) child marriage.

This study provides field data collected in Sierra Leone on several topics related to sexual and reproductive health. The topics considered are sexual and gender-based violence (SGBV), FP, child, early and forced marriage (CEFM), and FGM. The survey’s objective was to provide a picture of the norms related to those topics among the population, both men and women of various ages. Knowing what the perceptions related to sexual and reproductive health are allows us to identify the target groups (e.g., who needs to be convinced to change), who is supporting of healthy behaviors (i.e., who could have a role in the program as peer supporters) and what are the (false)-beliefs, values, and perceptions of the population that need to be addressed to build effective interventions.

A number of existing studies examined the questions of beliefs in the communities and norms related to those thematics. Steinhaus et al. (2019) studied social norms related to child marriage among decision-makers of young girls in Malawi. They showed that despite a low median age of marriage in the country, the perception of what others expect, measured with the agreement to the sentence “Most people in this community expect girls to marry before the age of 18,” was around 50%, that is, lower than it could have been expected. In South Sudan, Scott et al. (2013) assessed men’s and women’s attitudes toward sexual relationships and reproductive health. Respondents indicated their level of agreement with propositions, such as “it is a woman’s responsibility to avoid getting pregnant” or “it would be outrageous for a wife to ask her husband to use a condom.” The results suggest that gender inequitable norms are majoritarian and that this is the case among both sexes. Similarly, Nalukwago et al. (2019) assessed perceptions of gender norms in Uganda and showed that on average both adolescent girls and boys share gender norms, for example, 56% of girls and 58% of boys agree/partly agree that a woman should tolerate violence to keep the family together, and 64% of girls and 66% of boys considering that it is a woman’s responsibility to avoid getting pregnant. This study also show that norms are associated with sexual behaviors of the adolescents (e.g., contraception use). In the Philippines, a study showed that the level of awareness among students on several topics, such as family planning, prevention of abortion, maternal and child health, and prevention of reproductive tract infections varies, with some topics (e.g., that infertility can result from reproductive tract infection, and knowledge about some methods of family planning) having a mean level around 3 on a scale from 1 to 5, suggesting a need to intervene (Asio, 2019).

Existing studies mainly used Likert scale agreement with statements to measure norms and perceptions related to sexual and reproductive health. However, authors discussed the idea that the Likert scale is not necessarily optimal. For example, elements of the format, such as the ascending or descending order of the response options, influence the answers (e.g., Chyung et al., 2018). Acquiescence bias has also been highlighted about Likert scales, and authors suggested that semantic differential scales reduced the bias (Friborg et al., 2006). More specific to our study, Flaskerud (2012) reported several cases where participants of non-western origin had difficulties choosing one option among the scale and instead answered “yes” or “no” to each degree of the scale. In addition, other researchers identified cultural differences in the style of answering Likert scales (Lipnevich et al., 2011; He and van de Vijver, 2013). In this study, we use hypothetical scenarios, also called Situational Judgment Tests (SJTs), to grasp the norms and beliefs related to sexual and reproductive health in Sierra Leone.

Situational Judgment Tests have been chiefly used in education and occupational psychology (for school admissions and employee selection notably) to measure attributes, such as leadership and interpersonal skills (Christian et al., 2010). A scenario is presented to the person, and they have to indicate their typical response among the response possibilities. Arguments for SJTs are that they are less prone to bias associated with self-report (Lipnevich et al., 2013), such as “faking” or the practice of respondents trying to give an answer that they suspect the enumerator will think is the best choice. To our knowledge, the only application of SJTs to health behaviors is a paper by Heininger et al. (2021) on hand-hygiene competence. However, it remains a measure of skills, while in our study, we are interested in measuring the respondent perception of what they would do in a situation. We were also aiming to find a tool that would help us to specifically identify some of the underlying norms in a community disaggregated by age, sex, and other factors to help us understand where barriers existed and how to best address these in our health promotion work. The way the SJTs used in this study were developed is explained in detail in Appendix A.

The SJT tool was integrated into a household survey being used as a baseline survey for the BRIDGE project in Sierra Leone. The BRIDGE project is implemented by Sierra Leone Red Cross and supported by Finnish Red Cross and Icelandic Red Cross in six districts—Bo, Bonthe, Kenema, Kono, Moyamba, and Pujehun.

The paper has two goals: Report descriptive data on norms associated with SGBV, FP, CEFM, and FGM among different population groups (e.g., sex, age, and disability), which will benefit practitioners and policymakers by providing insights to build interventions. The second goal is to validate the use of SJTs in measuring hypothetical intentions/norms by comparing with other data, such as knowledge (e.g., for FP SJT: knowledge of a place where to obtain a method of family planning) and related behaviors (e.g., for SGBV SJT: self-report action when being witness of violence). Developing a new measurement method for norms and beliefs would help future interventions tailored to the target population-specific needs.

## Materials and Methods

### Research Design and Data Gathering Procedure

The study is a cross-sectional survey, conducted in Sierra Leone. Thirty villages were randomly selected from 62 villages where the project would be implemented in six districts, with five villages being selected from each district. Per each of the six districts, four enumerators were trained in the survey and assigned a team leader who was also present at the training. Team leaders and enumerators went to one village per day. Each enumerator was asked to collect at least five surveys per day. Teams traveled within their district for 5 days.

Among the villages, households were randomly selected, and potential respondents were visited in their homes to see if they were willing to participate. The data was collected using KoBo Toolbox software, all enumerators used their own smartphones loaded with the tested survey.

### Ethics

Potential respondents were informed of the institutional affiliation of the enumerator (the Sierra Leone Red Cross). In accordance with the Declaration of Helsinki, the enumerator made clear that participation is voluntary and that participants could refuse to answer any questions and end the survey at any time. No information bearing the name or identity of the person was collected, and participants were informed that the answers to all questions would remain strictly confidential. Participants were asked if they agreed to participate. The enumerator proceeded if they agreed for the interview to begin; otherwise, the experimenter thanked the person for their time and moved to interview the next person.

Data were collected as part of regular program monitoring; thus, ethics approval was not sought by the organization implementing the program and leading the monitoring and evaluation.

### Population and Sampling

The sample is composed of 566 responses. The minimum age is 18, and the maximum is 100, with the average at 38 years old (*SD* = 14; mode = 35). 56.4% are female, 43.3% male, and for 0.4%, the information is missing. The respondents are from various country districts: 17% from Bo, 17.7% from Bonthe, 12.5% from Kenema, 17.5% from Kono, 17.7% from Moyamba, and 17.7% from Pujehun.

### Instrument

The household survey covered basic demographic questions—including those enabling disaggregation by sex, age, and disability as well as questions on health, water, sanitation, and hygiene, disaster preparedness, and livelihoods.

#### Disability Measure

The level of disability was measured with the Washington Group Short Set of Disability Questions. The items measure difficulty seeing, even if wearing glasses; difficulty hearing, even if using a hearing aid; difficulty walking or climbing steps; difficulty remembering or concentrating; difficulty with self-case, such as washing all over or dressing; and difficulty communicating (understanding or being understood). For each item, response options range from “no, no difficulty”/“yes, some difficulty”/“yes, a lot of difficulty”/“cannot do at all.” Several severity cutoff are suggested by Mont (2006), ranging from the broadest definition to a disability to the most limited definition. For this research, we set the cutoff at having one domain or less with difficulties vs. more than one domain with difficulties.

#### Situational Judgment Tests

Both project staff at national and regional levels and locally recruited enumerators reviewed the SJTs. Together they agreed upon basic changes either to improve interpretation of the question in local languages or to make the options more relevant to context, for example, using foods regularly available and consumed in the nutrition SJT (not presented in this paper).

Enumerator training included a review of all SJTs. Enumerators had to translate the questions, written in English, into their local languages in real time. The Sierra Leone team focused on ensuring consistency of understanding and delivery by enumerators. During the training, each SJT was rehearsed first as a role play in front of the entire group, followed by discussion between all enumerators on how to capture the questions and responses. Enumerators then rehearsed each question in partnered role play with team leaders observing and mentoring. Where issues with meaning, context, and language were identified changes were made directly into the Kobo Toolbox survey and then re-tested with the enumerators.

Ten^1^ SJTs were presented to respondents: three SJTs were about FGM (two for female respondents, one for male respondents), three SJTs were related to CEFM, two related to FP-adolescent pregnancy, and two about SGBV. Skip logic was programmed in the survey tool in Kobo Toolbox to ensure SJTs were fed appropriately to either male of female participants. Due to time limitations related to travel logistics and security and the overall length of the survey with other questions from the baseline survey, skip logic was also introduced to ensure that each respondent only received a smaller subset of the 10 possible SJTs rather than all of them. Skip logic was not based on a respondents answer to any question, but rather on the order in which they were interviewed by the enumerator that day. So while the first person an enumerator interviews in a village may receive an SJT on a particular topic, a subsequent person interviewed in that same village would receive an SJT on a different topic. This leads to different respondent sample size for each SJTs.

The response options contain one action that is the most appropriate for the question asked in that situation (coded to have the highest value, that is, 5), one or two actions that are somewhat appropriate (coded to an intermediate value, between 2 and 4), and one or two actions that would be inappropriate for the question asked in that situation (coded to the lowest value, that is, 1). Each response option is intended to be logically possible for the specific scenario. Respondents were instructed to choose the option that is closest to what they would do. SJTs and the response options and coding are presented in Table 1.

**Table 1:** Description of the situational judgment tests (SJTs) and their response options.

Sexual and gender-based violence SJTs					
**SGBV1:** Imagine that your boyfriend, Patrick, has recently gotten an excellent job in the capital city with a reliable organization. Your sick mother is very pleased and tells you that the whole family will be well cared for once you marry him. The stress Patrick is feeling is quite high and he sometimes takes it on you with slaps to your face or punching you in the back. Which of the following are you most likely to do?					
Tell Patrick you no longer will be his girlfriend (5)	Tell Patrick he must stop hitting you or you will not marry him (4)	Tell your mother that he is abusive so she will tell him to stop (3)		Hit Patrick back in the hopes that he will stop (2)	Put up with his abuse and hope it gets better (1)
**SGBV2:** Imagine that you see a man beating his wife at the market, shouting that she throws away his money. She is crouched down and protecting her head while the crowd watches him hit her with a strip of leather. Her small child is crying and pulling at his father to stop but he just pushes him away. The man is older and the wife is about your age. Which of the following are you lost likely to do?					
Comfort the child in the hopes that the father will realize what he is doing and stop (6)	Call the police or guards to stop it (5)	Tell the man you will report them to the police if he does not stop (4)	Shout at the man to stop (3)	Stand and watch in the hopes that someone does something (2)	Leave them to their business and walk on (1)
*Family planning SJTs*					
**FP1:** Imagine that your oldest brother, Musa, is planning to marry one of your friends, Fatmata. Fatmata said she wants to wait to have babies until she has finished school. She wants to use contraceptives and asks you what she should do. Which of the following are you most likely to do?					
Tell Fatmata that she should get contraceptive pamphlets from the clinic and talk with Musa about the options they have as a couple (6)	Tell Fatmata to tell Musa that she does not want to have babies right away and he should respect that. Tell her that you will tell Musa that he should respect women (5)	Tell Fatmata to just quietly go to the clinic and get the injectable that will keep the babies from coming for 3 months at a time. Musa does not need to know (4)	Tell Fatmata to abstain from sex (3)	Tell Fatmata that you plan to tell your and her father and mother that she plans to use contraceptives. It is her duty to have babies (2)	Tell Fatmata to just urinate or douche with vinegar after sex to keep from becoming pregnant (1)
**FP2:** Imagine that your boyfriend, Samuel, wants to have sex. He says that he knows that he will marry you when he finishes school, so it is your duty to have sex with him now, to ensure that he loses his virginity to you. You tell him that you will do so but only if you both use contraceptives. Your boyfriend says that he knows that you cannot get pregnant the first time and a condom is not necessary because you cannot have an STI if you are both virgins. Which of the following would you be most likely to do?					
Tell Samuel that you will only have sex if he wears a condom and you use another form of protection (5)	Tell Samuel that his knowledge of reproductive health is poor and you will both go to clinic to get the accurate information (4)	Tell Samuel that you will not have sex before marriage (3)		Tell Samuel that you are not so sure that what he says is true, but you trust him and will do as he asks (2)	Tell Samuel that you agree that having sex the first time is safe, so you are happy to do so (1)
*Adolescent pregnancy—child early and forced marriage SJTs*					
**AP-CEFM1:** Imagine that you are 16 years old, the oldest girl in your family and your mother is 16 years your senior. People say you look like sisters more than like mother and daughter. Your mother cannot read well because she left school when she fell pregnant with you. You want to stay in school which vexes her. She tells you to just make a baby with Momodu and start your life—it was a good enough life for her. Which of the following would you be most likely to do?					
You tell your mother that you want to go to university and get a good job (5)	You tell your mother that you do not want to have babies when you are not married (4)	You tell your mother that you do not like Momodu enough to make babies with him (3)		You tell your mother that you will consider this, but you secretly plan on staying in school (2)	You tell your mother that you do not want babies at all just to anger her (1)
**AP-CEFM2:** Imagine that you are in your third year of secondary school and you want to continue studying to become a solicitor. Your mother says that women make for bad solicitors because they are too emotional and she cannot wait so long for you to either leave the house or start earning money to help the family. Your friends are all getting married or are married and pregnant with their first babies. You just learned from your teacher how many years of school is required to become a solicitor and the fees for law school. You are discouraged. Which of the following would you be most likely to do?					
Ask the teacher for help in finding scholarships to help you attend university (5)	Tell your mother how much money solicitors can earn and that by ensuring that you get into law school and graduate, you will be able to support her for life (4)	Stay in school and choose another career that requires less formal education (3)		Give up your plan to go to law school and quit school for a job that will pay money now (2)	Give up your plan to go to law school and quit school to get married to your boyfriend (1)
**AP-CEFM3:** Imagine that your father has informed you that he cannot afford to feed so many mouths in the house. As the oldest daughter, he has found a husband for you to marry to remove some of the financial strain and for you to do your duty to the family. The man that your father has chosen is much older than you, has many children from his previous wife who dies 2 years ago and was cruel to his wife. Which of the following would you be most likely to do?					
Tell your father that you refuse to marry and that you will stay in school so that you will 1 day have a good-paying job (5)	Tell your father that the man was cruel to his previous wife and hope that your father shows you mercy and chooses another man for you to marry (4)	Tell your father that you will not marry him and will go to the capital city to find work and send money home (3)		Tell your father that you will marry the man only if your father allows you to get the stick to keep from getting pregnant (2)	Tell your father that you will do as he asks and you agree to marry the man (1)
*Female genital mutilation SJTs*					
**FGM1:** Imagine that your mother was cut when she was eleven. She has always said that she would keep you, her own daughter from having to endure cutting. Your grandmother has said that she is upset that her own granddaughter remains uncut and has asked you to undergo circumcision to honor her. Which of the following would you be most likely to do?					
Tell your grandmother that she is very brave but that you will not be cut (5)	Tell your grandmother that you cannot disobey your mother (4)	Tell your grandmother that she is very brave but that you are afraid (3)		Tell your grandmother that you will do it but that you will choose the type of circumcision and the circumciser (2)	Tell your grandmother that you will submit to being cut (1)
**FGM2:** Imagine that you were cut when you were 12 years old. You still feel pain when you bleed each month and you have a difficult time making good friends, finding that you are anxious and find it hard to trust people. You can still see your grandmother between your legs when she hurt you so. You are talking with a small group of women about when they have children—whether they will allow their daughters to be cut. One of the women says that she will “definitely cut” her daughter, saying it is tradition and that since she was cut, her daughters must also be cut. Which of the following would you be most likely to do?					
Explain the physical and psychosocial problems that many girls and women suffer from cutting and explain that you, too, still suffer these effects (5)	Explain the physical and psychosocial problems that many girls and women suffer from cutting but do not mention that you also suffer these (4)	Disagree with the woman publicly, saying that it is barbarian (3)		Agree with the woman publicly just to get her to stop talking about it (2)	Agree that traditions are strong around cutting and that women should suffer to maintain these customs (1)
**FGM3:** Imagine that you are a man in your early 30s who is eager to get married and start a family. Your father tells you that a good woman is one who has been cut and whose purity is assured for your wedding night. He tells you that a good woman is cut to receive you on your wedding night and you will know no other man has been able to take what is rightfully yours. You know that your girlfriend has not been cut as severely as your father thinks is proper for a woman. Which of the following would you be most likely to do?					
Tell your father that you do not care about these matters—times are changing and traditions must change so that women are no longer expected to be cut (5)	Tell your father that you are sure that her purity is secured and change the topic (4)	Tell your father that your girlfriend was not cut as severely as your father indicates and ask what you should do (3)		Tell your father that you will find a woman to marry who has been cut in such a way to ensure purity (2)	Tell your father that you will ask your girlfriend to undergo a more severe cutting so that you can marry her (1)

#### Validation Items

##### Self-Report Reaction to Violence (for SGBV SJTs)

Two items measured the self-reported reaction to violence: “If you saw or heard someone being sexually violent against another person, what immediate action could you take?,” no response option was read aloud, but the response given by the participant was classified by the enumerator in one of the following options: *Get the person being hurt to safety; Get help immediately; Speak up to bring attention to the violence; Make it clear to the inflictor that violence is unacceptable and must stop immediately; Talk to someone else in the home or community that can help; Other (please specify); Do not know*. Except for the “Do not know” answer, all responses mentioned by the participant were summed to compose its score for this question. The average score for this item is 1.52 (*SD* = 0.87).

The second item measuring reaction to violence is: “If a person tells you they are being hurt by violence, what can you do to help the person?.” Similarly, the response given by participants were coded by the enumerator in one of the following categories: *Listen to the person and show empathy; Comfort the person; Take the person to a safe place; Know the community resources and support system; If it involves a child, report the violence to a helping source in the community, Other; Do not know*. The score was computed by summing all responses except “Do not know.” Average score for this item is 1.56 (*SD* = 0.83).

##### Knowledge of Where to Obtain Contraception (for FP SJTs)

One item measured the knowledge about where to obtain contraception: “Do you know of a place where you could obtain a method of child spacing/family planning”? No response option was read aloud by the enumerator. They recorded the responses given by participants in one of the following possibilities: *Hospital; Public Health Unit; Health Centre, Community Health Center, Marie Stopes; Community Health Worker/Pharmacy; Shop; Friend/relative; Other* (*please specify*). If at least one place was mentioned, response is coded as 1, while if the respondent could not mention a place where to obtain FP, they received the score of 0. Average score for this item is 0.95 (*SD* = 0.22).

### Statistical Treatment/Analysis

#### Descriptives of Stigma-Sensitive Norms on the Four Topics (SGBV, FP/AP, CEFM, and FGM), General Overview, and Crossed by Characteristics (Age/Gender/Handicap)

Statistical differences are tested with nonparametric rank tests (Field, 2018; Gibbons and Chakraborti, 2020; two tails tests, significance threshold < 0.05), respectively for disability and gender (two groups- > Mann–Whitney test), and age (more than two groups- > Kruskal–Wallis) because answers to the SJTs are not an interval measurement. For some SJTs, the number of participants in the category “with more than one domain with difficulties” was much lower than in the group “one domain or less with difficulties.” Analysis on disability was not performed if there were less than 10 respondents in each group.

#### Validation of SJTs Answers With Other Types of Data

Construct validity of SJT is tested with Spearman’s rank correlation by assessing the association between the SJT’s answers and other measures in the survey that are judged relevant according to the topic of the SJT. Unfortunately, for some SJTs (those on FGM in particular), there was no “objective” measure to do such analysis. They are thus not included in this part of the paper.

## Results

### Descriptives of Stigma-Sensitive Norms on the Four Topics (SGBV, FP/AP, CEFM, and FGM), General Overview and Crossed by Characteristics (Age/Gender/Handicap)

#### Sexual and Gender-Based Violence SJTs

**SGBV1—Imagine that your boyfriend, Patrick, has recently gotten an excellent job in the capital city with a reliable organization. Your sick mother is very pleased and tells you that the whole family will be well cared for once you marry him. The stress Patrick is feeling is quite high and he sometimes takes it on you with slaps to your face or punching you in the back.**

Responses to the SJT SGBV1 (*N* = 115) were the following: 29.6% of respondents chose the answer “Tell Patrick you no longer will be his girlfriend” (coded 5). A similar share of respondents chose answers “Tell Patrick he must stop hitting you or you will not marry him” (27.8%, coded 4) and “Tell your mother that he is abusive so she will tell him to stop” (30.4%, coded 3). Very few respondents choose the answer “Hit Patrick back in the hopes that he will stop” (5.2%, coded 2) or the answer “Put up with his abuse and hope it gets better” (6.9%, coded 1).

**Figure 1:**
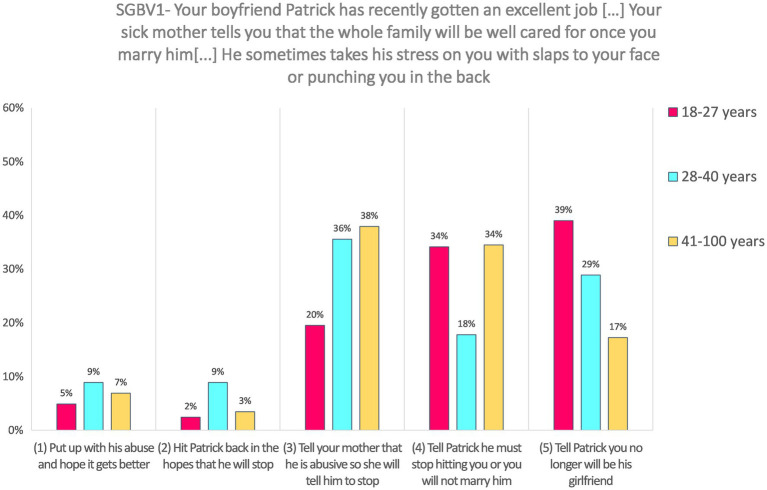
Answers to SGBV1 according to age categories.

Answers on SGBV1 according to age were tested using Kruskal–Wallis test, which shows a non-significant but marginal effect, *H*(2) = 5.66, *p* = 0.059. Results are presented in Figure 1. Descriptive results suggest that younger respondents choose more the highest coded choice (i.e., the most appropriate in our coding), while referring to the mother seems more frequent among middle-aged and older participants.

Response on this SJT was not analyzed according to gender because it was presented to female respondents only.

Responses to SGBV1 did not significantly varied according to the disability level, as Mann–Whitney test shows, *U* = 833.50, *z* = −0.76, *p* = 0.450, and *r* = −0.07. Both groups (respondents with one domain or less with difficulties and respondents with more than one domain with difficulties) have a median value = 4.

**SGBV2—Imagine that you see a man beating his wife at the market, shouting that she throws away his money. She is crouched down and protecting her head while the crowd watches him hit her with a strip of leather. Her small child is crying and pulling at his father to stop but he just pushes him away. The man is older and the wife is about your age.**

Responses to the SJT SGBV2 (*N* = 217) were around a quarter of respondents for each of the four highest coded choices: 20.3% for the answer “Comfort the child in the hope that the father will realize what he is doing and stop” (coded 6); 25.8% “Call the police or guards to stop it” (coded 5); 24.4% “Tell the man you will report them to the police if he does not stop” (coded 4), and 25.3% “Shout at the man to stop” (coded 3). On the other hand, nearly no respondent chose the two lowest ranked answers, “Stand and watch in the hope that someone does something” (2.3%, coded 2), or the answer “Leave them to their business and walk on” (1.8%, coded 1).

Answers on SGBV2 did not differ according to age, *H*(2) = 4.32, *p* = 0.115, neither to according to gender, *U* = 5858.00, *z* = 0.15, *p* = 0.881, and *r* = 0.01. Both male and female respondents have a median value = 4 (i.e., “Tell the man you will report them to the police if he does not stop”). Responses to SGBV2 also did not significantly varied according to the disability level, *U* = 4514.00, *z* = 0.295, *p* = 0.768, and *r* = 0.02. Both groups (respondents with one domain or less with difficulties and respondents with more than one domain with difficulties) have a median value = 4.

#### Family Planning SJTs

**FP1—Imagine that your oldest brother, Musa, is planning to marry one of your friends, Fatmata. Fatmata said she wants to wait to have babies until she has finished school. She wants to use contraceptives and asks you what she should do.**

Responses to the SJT FP1 (*N* = 235) were the following: 11.1% of respondents chose the answer “Tell Fatmata that she should get contraceptive pamphlets from the clinic and talk with Musa about the options they have as a couple” (coded 6). Twice more respondents chose the answer “Tell Fatmata to tell Musa that she does not want to have babies right away and he should respect that. Tell her that you will tell Musa that he should respect women” (22.1%, coded 5), and three times more chose the answer “Tell Fatmata to just quietly go to the clinic and get the injectable that will keep the babies from coming for 3 months at a time. Musa does not need to know” (33.6%, coded 4). Around 8.5% chose the answer “Tell Fatmata to abstain from sex” (coded 3), and 21.7% the answer coded 2 “Tell Fatmata that you plan to tell your and her father and mother that she plans to use contraceptives. It is her duty to have babies.” A very small percentage (3%) chose the option coded 1, “Tell Fatmata to just urinate or douche with vinegar after sex to keep from becoming pregnant.”

Answers on FP1 did not vary according to age *H*(2) = 0.08, *p* = 0.963, neither according to gender, *U* = 6437.00, *z* = −0.24, *p* = 0.808, and *r* = −0.02. Both male and female respondents have a median value = 3 (i.e., “Tell her to just quietly go to the clinic and get the injectable that will keep the babies from coming”). Responses to FP did not significantly vary according to the disability level, although the *p*-value is close to the significance threshold, *U* = 6115.00, *z* = 1.90, *p* = 0.057, and *r* = 0.12. Both groups (respondents with one domain or less with difficulties and respondents with more than one domain with difficulties) have a median value = 4.

**FP2—Imagine that your boyfriend, Samuel, wants to have sex. He says that he knows that he will marry you when he finishes school, so it is your duty to have sex with him now, to ensure that he loses his virginity to you. You tell him that you will do so but only if you both use contraceptives. Your boyfriend says that he knows that you cannot get pregnant the first time and a condom is not necessary because you cannot have an sexually transmitted infection (STI) if you are both virgins.**

Responses to the SJT FP2 (*N* = 59) were frequent for the highest coded choice: 35.6% answered “Tell Samuel that you will only have sex if he wears a condom and you use another form of protection” (coded 5). Around 8.5% chose the option “Tell Samuel that his knowledge of reproductive health is poor and you will both go to clinic to get the accurate information” (coded 4). The majority (37.3%) choose the third option, “Tell Samuel that you will not have sex before marriage” (coded 3). Around 13.6% chose the option “Tell Samuel that you are not so sure that what he says is true, but you trust him and will do as he asks” (coded 2), and a small minority answered “Tell Samuel that you agree that having sex the first time is safe, so you are happy to do so” (5.1%, coded 1).

**Figure 2:**
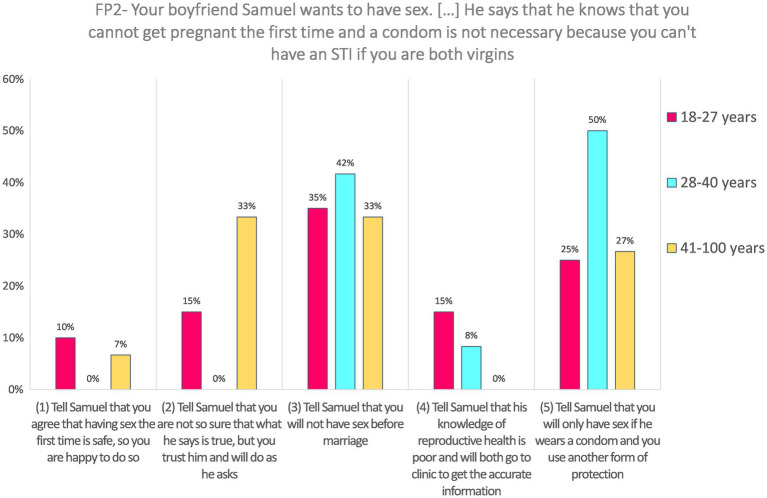
Answers to FP2 according to age categories.

Responses to this SJT were not analyzed according to gender because it was presented to female respondents only, neither according to disability level. Answers on FP2 significantly differed according to age, *H*(2) = 7.63, *p* = 0.022. Pairwise comparisons with adjusted *p*-values showed that the difference occurred mainly between the oldest and the middle-aged group (*p* = 0.032, *r* = 0.41), the latter ones providing higher-coded answers than the former. The comparison between the youngest and the middle-aged group is not significant, although the effect size is not negligible (*p* = 0.126, *r* = −0.31), neither the comparison between the oldest and the youngest (*p* = 1.00, *r* = 0.11). Results are presented in Figure 2.

#### Adolescent Pregnancy—Child Early and Forced Marriage SJTs

**AP-CEFM1—Imagine that you are 16 years old, the oldest girl in your family and your mother is 16 years your senior. People say you look like sisters more than like mother and daughter. Your mother cannot read well because she left school when she fell pregnant with you. You want to stay in school which vexes her. She tells you to just make a baby with Momodu and start your life—it was a good enough life for her.**

Responses to the SJT AP-CEFM1 (*N* = 79) were majoritarian for the highest coded choice: 51.9% for the answer “You tell your mother than you want to go to university and get a good job” (coded 5). Around 13.9% chose the option “You tell your mother that you do not want to have babies when you are not married” (coded 4); 10.1% “You tell your mother that you do not like Momodu enough to make babies with him” (coded 3), and 19.0% “You tell your mother that you will consider this, but you secretly plan on staying in school” (coded 2). Very few respondents choose the lowest ranked answer, “You tell your mother that you do not want babies at all just to anger her” (5.1%, coded 1).

Answers on AP-CEFM1 did not differ according to age, *H*(2) = 2.61, *p* = 0.271. Response on this SJT was not analyzed according to gender because it was presented to female respondents only. Responses to AP-CEFM1 did not significantly varied according to the disability level, *U* = 599.00, *z* = 0.36, *p* = 0.718, and *r* = 0.04. It is, however, important to note that only 19 respondents to this SJT had difficulties in more than one domain; thus, the statistical power might be low. Respondents with one domain or less with difficulties have a median value of 4.5, while respondents with more than one domain with difficulties have a median value = 5.

**AP-CEFM2—Imagine that you are in your third year of secondary school and you want to continue studying to become a solicitor. Your mother says that women make for bad solicitors because they are too emotional and she cannot wait so long for you to either leave the house or start earning money to help the family. Your friends are all getting married or are married and pregnant with their first babies. You just learned from your teacher how many years of school is required to become a solicitor and the fees for law school. You are discouraged.**

Responses to the SJT AP-CEFM2 (*N* = 55) were most frequent for the highest coded choice: 41.8% answered, “Ask the teacher for help in finding scholarships to help you attend university” (coded 5). Around 34.5% chose the option “Tell your mother how much money solicitors can earn and that by ensuring that you get into law school and graduate, you will be able to support her for life” (coded 4); 12.7% “Stay in school and choose another career that requires less formal education” (coded 3). Few respondents chose the two lowest coded choices: 7.3% “Give up your plan to go to law school and quit school for a job that will pay money now” (coded 2), and “Give up your plan to go to law school and quit school to get married to your boyfriend” (3.6%, coded 1).

Response on this SJT was not analyzed according to gender because it was presented to female respondents only. Answers on AP-CEFM2 did not differ according to age, *H*(2) = 2.24, *p* = 0.327, neither according to the disability level, *U* = 179.50, *z* = −1.30, *p* = 0.193, and *r* = −0.18. Similarly to AP-CEFM1, the statistical power might be low because only 11 respondents to AP-CEFM2 had difficulties in more than one domain. Both respondents with one domain or less with difficulties and those with more than one domain with difficulties have a median value = 4 (i.e., “Tell your mother how much money solicitors can earn”).

**AP-CEFM3—Imagine that your father has informed you that he cannot afford to feed so many mouths in the house. As the oldest daughter, he has found a husband for you to marry to remove some of the financial strain and for you to do your duty to the family. The man that your father has chosen is much older than you, has many children from his previous wife who dies 2 years ago, and was cruel to his wife.**

Responses to the SJT AP-CEFM3 (*N* = 56) were most frequent for the middle answer (coded 3) “Tell your father that you will not marry him and will go to the capital city to find work and send money home,” 33.9%; followed by the highest coded answer (5) “Tell your father that you refuse to marry and that you will stay in school so that you will 1 day have a good-paying job” chosen by 25% of respondents, and the lowest coded answer (1) “Tell your father that you will do as he asks and you agree to marry the man,” chosen by 23.2%. The rest of the respondents choose equally the second lowest response option (2) “Tell your father that you will marry the man only if your father allows you to get the stick to keep from getting pregnant,” and the response coded 4 “Tell your father that the man was cruel to his previous wife and hope that your father shows you mercy and chooses another man for you to marry,” each chosen by 8.9% of respondents.

Response on this SJT was not analyzed according to gender because it was presented to female respondents only, and neither on disability level. Answers on AP-CEFM3 did not differ according to age, *H*(2) = 1.46, *p* = 0.483.

#### Female Genital Mutilation SJTs

**FGM1—Imagine that your mother was cut when she was 11. She has always said that she would keep you, her own daughter from having to endure cutting. Your grandmother has said that she is upset that her own granddaughter remains uncut and has asked you to undergo circumcision to honor her.**

Responses to the SJT FGM1 (*N* = 55) were not very frequent for the highest coded choice: only 12.7% answered “Tell your grandmother that she is very brave but that you will not be cut” (coded 5). A bigger share of respondents (30.9%) chose the option “Tell your grandmother that you cannot disobey your mother” (coded 4). Around 18.2% choose the third option, “Tell your grandmother that she is very brave but that you are afraid” (coded 3). Around 7.3% chose the option “Tell your grandmother that you will do it but that you will choose the type of circumcision and the circumciser” (coded 2), and around a third (30.9%) answered “Tell your grandmother that you will submit to being cut” (coded 1).

Responses to this SJT were not analyzed according to gender because it was presented to female respondents only, neither according to disability level. Answers on FGM1 did not differ according to age, *H*(2) = 2.50, *p* = 0.287.

**FGM2—Imagine that you were cut when you were 12 years old. You still feel pain when you bleed each month and you have a difficult time making good friends, finding that you are anxious and find it hard to trust people. You can still see your grandmother between your legs when she hurt you so. You are talking with a small group of women about when they have children—whether they will allow their daughters to be cut. One of the women says that she will “definitely cut” her daughter, saying it is tradition and that since she was cut, her daughters must also be cut.**

Responses to the SJT FGM2 (*N* = 59) were not very frequent for the highest coded choice: only 11.9% answered “Explain the physical and psychosocial problems that many girls and women suffer from cutting and explain that you, too, still suffer these effects” (coded 5). No participants chose the fourth option “Explain the physical and psychosocial problems that many girls and women suffer from cutting, but do not mention that you also suffer these” (coded 4). Around 16.9% choose the third option, “Disagree with your friend publicly, saying that it is barbarian” (coded 3). The majority of respondents (42.4%) chose the option “Agree with the friend publicly just to get her to stop talking about it” (coded 2), and nearly a third (28.8%) answered “Agree that traditions are strong around cutting and that women should suffer to maintain these customs” (coded 1).

**Figure 3:**
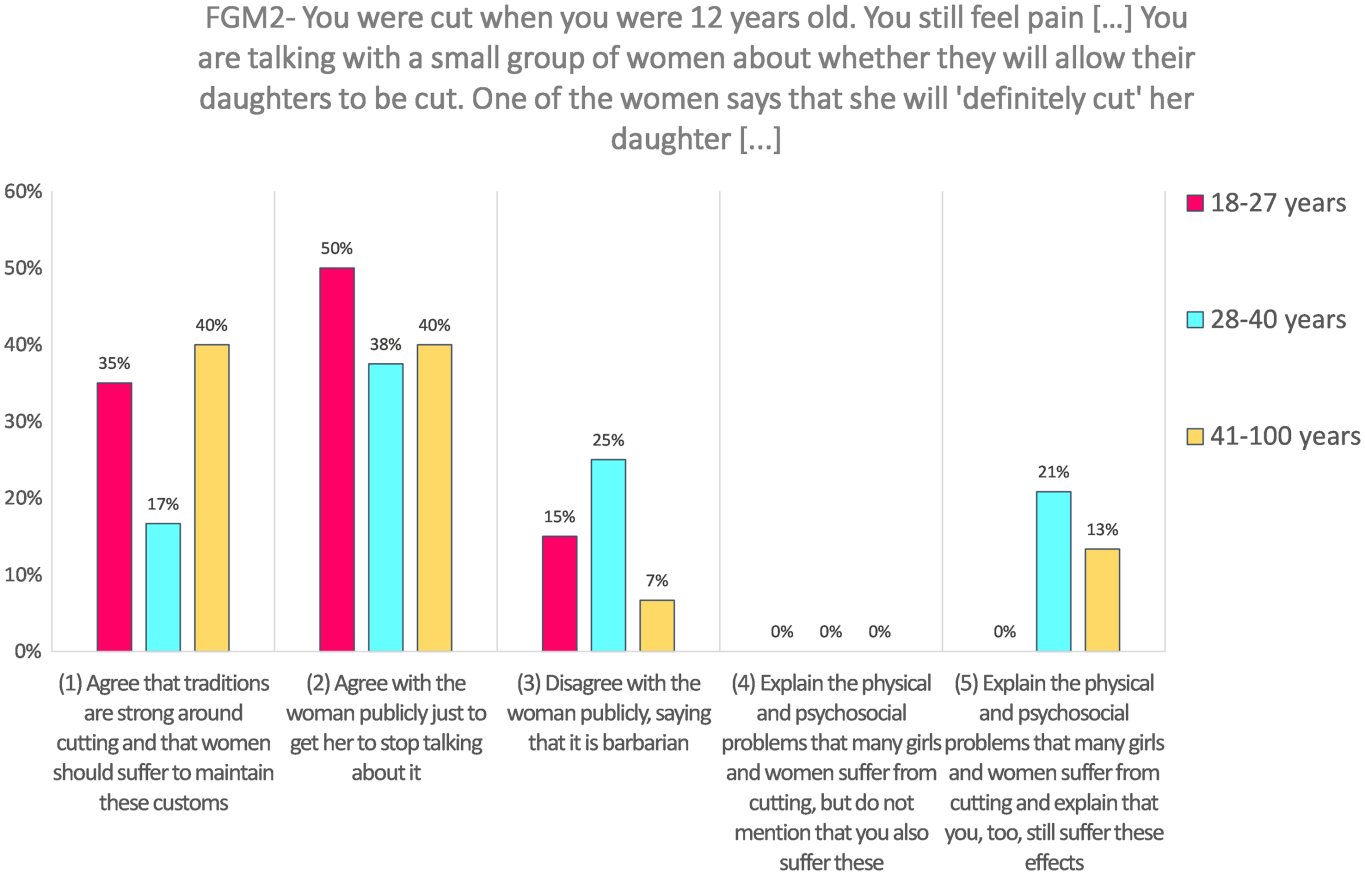
Answers to FGM2 according to age categories.

Responses to this SJT were not analyzed according to gender because it was presented to female respondents only, neither according to disability level. The effect of age is significant, *H*(2) = 6.10, *p* = 0.047. Pairwise comparisons with adjusted *p*-values showed that the difference occurred mainly between the youngest and the middle-aged group (the latter providing higher-coded answers than the former), although after the adjustment, the *p*-value is not below the 5% threshold (*p* = 0.075, *r* = −0.34). The difference between the oldest and the middle-aged group is not significant (*p* = 0.179, *r* = 0.30), neither is the comparison between the youngest and the oldest (*p* = 1.00, *r* = −0.03). Results are presented in Figure 3.

**FGM3—Imagine that you are a man in your early 30s who is eager to get married and start a family. Your father tells you that a good woman is one who has been cut and whose purity is assured for your wedding night. He tells you that a good woman is cut to receive you on your wedding night and you will know no other man has been able to take what is rightfully yours. You know that your girlfriend has not been cut as severely as your father thinks is proper for a woman.**

Responses to the SJT FGM3 (*N* = 245) were not very frequent for the three highest coded choices: 18.0% answered “Tell your father that you do not care about these matters—times are changing and traditions must change so that women are no longer expected to be cut” (coded 5); 12.2% choose “Tell your father that you are sure that her purity is secured and change the topic” (coded 4), and 14.3% choose the third option, “Tell your father that your girlfriend was not cut as severely as your father indicates and ask what you should do” (coded 3). Nearly two-thirds of the respondents choose one of the two lowest coded options: 29% choose “Tell your father that you will find a woman to marry who has been cut in such a way to ensure purity” (coded 2), and 26.5% choose “Tell your father that you will ask your girlfriend to undergo a more severe cutting so that you can marry her” (coded 1).

**Figure 4:**
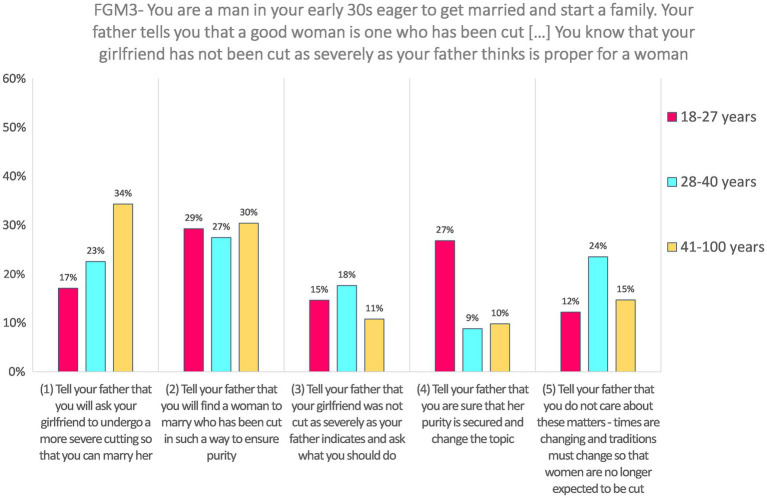
Answers to FGM3 according to age categories.

Response on this SJT was not analyzed according to gender because it was presented to male respondents only. The effect of age is statistically significant, *H*(2) = 6.51, *p* = 0.039. Pairwise comparisons with adjusted *p*-values showed that the difference occurred mainly between the oldest and the middle-aged group (the latter providing higher-coded answers than the former), although after the adjustment, the *p*-value is not below the 5% threshold (*p* = 0.076, *r* = 0.16). The difference between the oldest and the youngest group is not significant after the adjustment (*p* = 0.143, *r* = 0.17), neither is the comparison between the youngest and the middle-aged group (*p* = 1.00, *r* = 0.02). Results are presented in Figure 4.

Responses to FGM3 did not significantly varied according to the disability level, *U* = 6668.00, *z* = 0.688, *p* = 0.491, and *r* = 0.04. Both groups (respondents with one domain or less with difficulties and respondents with more than one domain with difficulties) have a median value = 2.

### Validation of SJTs Answers With Other Types of Data

#### Sexual and Gender-Based Violence SJTs

For SGBV vignettes, the SJTs answers are compared with self-report of action taken if one saw or heard someone being sexually violent against another person; and self-report of action if a person tells oneself that they are the victim of violence.

**SGBV1—Imagine that your boyfriend, Patrick, has recently gotten an excellent job in the capital city with a reliable organization. Your sick mother is very pleased and tells you that the whole family will be well cared for once you marry him. The stress Patrick is feeling is quite high and he sometimes takes it on you with slaps to your face or punching you in the back.**

**Figure 5:**
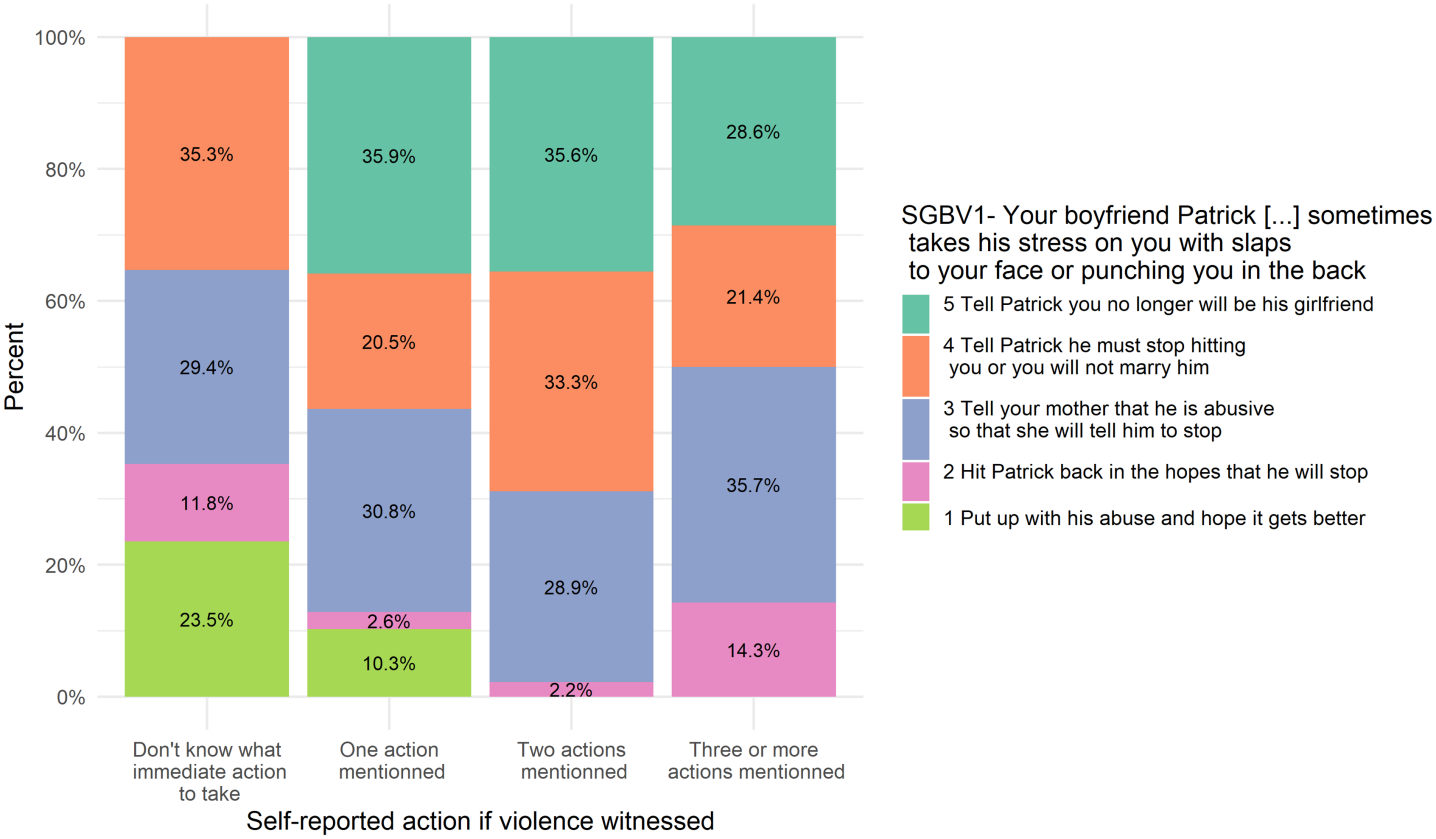
SGBV1 answers put in relation to self-reported action in case of witnessing violence.

Answers to SGBV1 are positively and significantly correlated with self-report action in case of seeing or hearing someone being sexually violent against another person, *r_s_* = 0.21, *p* = 0.026. Figure 5 illustrates the association between SJT’s answer and self-reported action.

On the contrary, answers to SGBV1 are not significantly correlated with self-report action if someone tells they are the victim of violence, *r_s_* = 0.15, *p* = 0.110.

**SGBV2—Imagine that you see a man beating his wife at the market, shouting that she throws away his money. She is crouched down and protecting her head while the crowd watches him hit her with a strip of leather. Her small child is crying and pulling at his father to stop but he just pushes him away. The man is older and the wife is about your age.**

**Figure 6:**
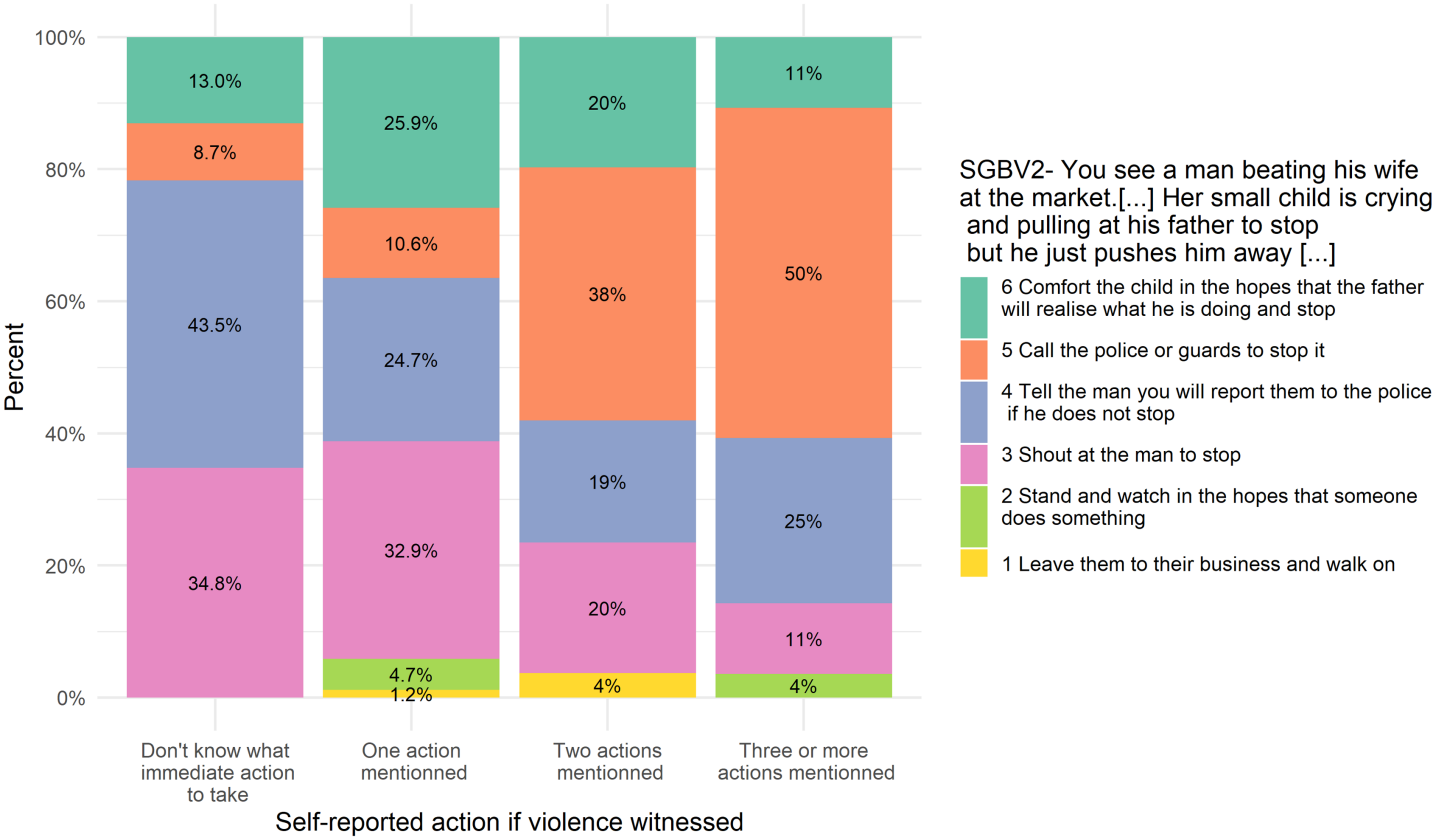
SGBV2 answers put in relation to self-reported action in case of witnessing violence.

Answers to SGBV2 are positively and significantly correlated with self-report action in case of seeing or hearing someone being sexually violent against another person, *r_s_* = 0.17, *p* = 0.012. Figure 6 illustrates the association between this SJT’s answer and self-reported action. Answers to SGBV2 are not significantly correlated with self-report action if someone tells they are the victim of violence, *r_s_* = 0.12, *p* = 0.085.

#### Family Planning SJTs

**FP1—Imagine that your oldest brother, Musa, is planning to marry one of your friends, Fatmata. Fatmata said she wants to wait to have babies until she has finished school. She wants to use contraceptives and asks you what she should do.**

**Figure 7:**
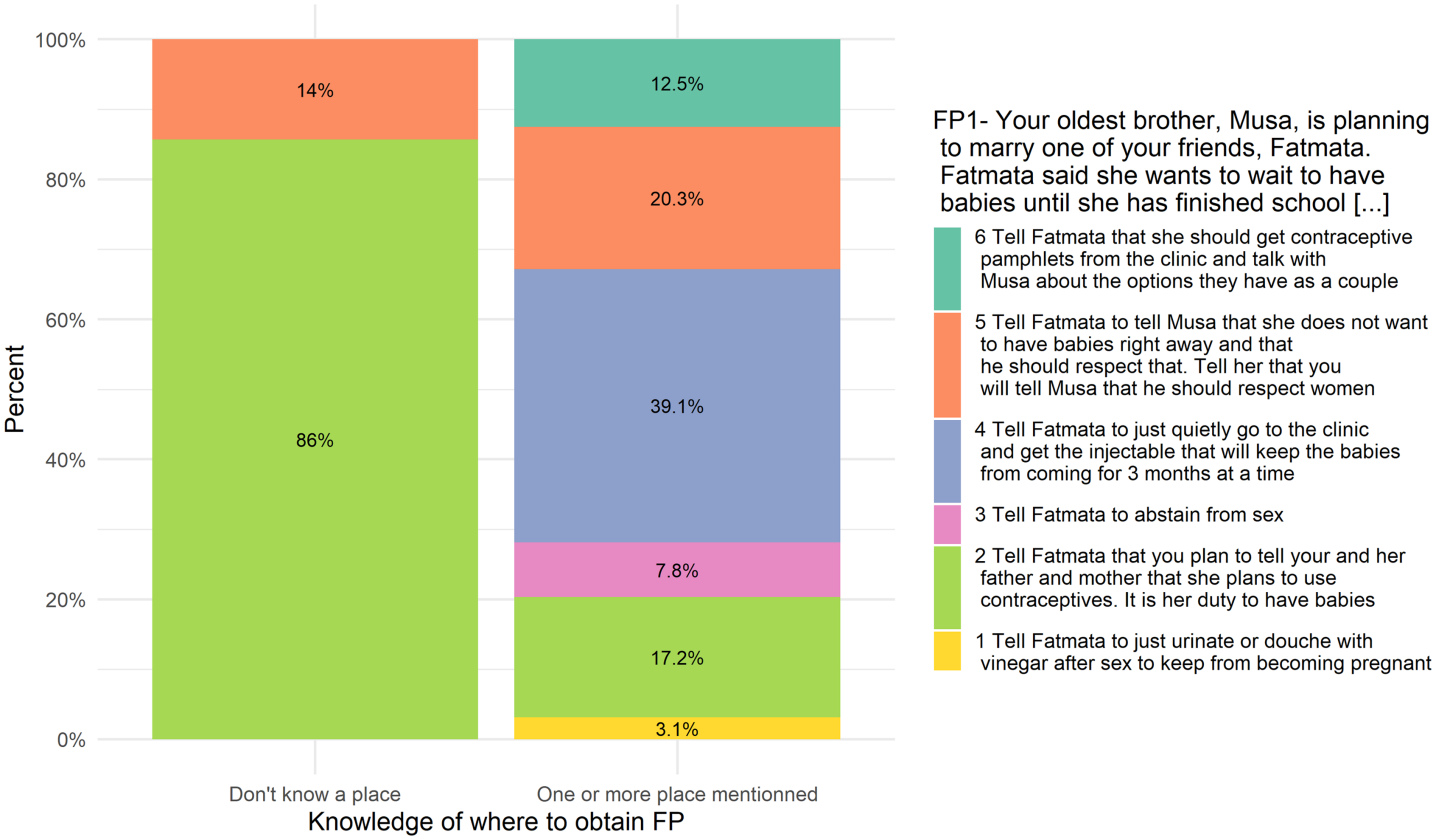
FP1 answers put in relation to self-reported knowledge about obtaining family planning.

Answers to FP1 are correlated with the knowledge of where to obtain a method of child spacing/family planning (i.e., zero places cited vs. one or more) *r_s_* = 0.23, *p* = 0.007 (see Figure 7). However, it has to be noted that very few people did not know even one place to obtain FP (eight respondents), so the correlation has to be interpreted cautiously.

**FP2—Imagine that your boyfriend, Samuel, wants to have sex. He says that he knows that he will marry you when he finishes school, so it is your duty to have sex with him now, to ensure that he loses his virginity to you. You tell him that you will do so but only if you both use contraceptives. Your boyfriend says that he knows that you cannot get pregnant the first time and a condom is not necessary because you cannot have an STI if you are both virgins.**

Answers to FP2 are not correlated with the knowledge of where to obtain a method of child spacing/family planning (i.e., zero places cited vs. one or more) *r_s_* = 0.01, *p* = 0.948.

## Discussion

This study aimed to achieve two things:

1. To describe the norms and beliefs related to several sexual and reproductive health topics using an innovative method of measurement, SJTs. Answers to the SJTs show that there is a margin of progress in terms of norms and beliefs, as the highest coded answer was not always the majoritarian response. FGM in particular seems to be a priority topic, as the answers to SJTs on FGM are worrisome. Relatively to FGM, SJTs on SGBV and CEFM had relatively high level of answers, expressing that the norms are stronger against those practices.

Two SJTs were presented to respondents of both gender (SGBV2 and FP1) but in both cases no significant difference was detected on the responses according to gender. This lack of difference might come as surprising; however, other studies showed that gender inequitable norms are often shared by both men and women (e.g., Scott et al., 2013; Nalukwago et al., 2019).

Age effect was sometimes detected but did not always go in the same direction. For FP2 and FGM3, the middle-aged group gave higher-coded answers than the older respondents, and in the same direction (but only marginally significant) on SGBV1 the younger gave higher-coded answers than the oldest group. On the contrary, a reversed-aged effect was found on FGM2, for which younger participants provided lower-coded answers than the middle-aged group. On the topic of FGM, it does suggest that younger female might be a target group for an intervention (based on FGM2 results), while in the case of male it is the older ones that could be targeted (based on FGM3 results).

The association between the answers and disability status was tested when possible, but in many occasions there were less than 10 respondents in the group with more than one domain with difficulties. No significant association was detected with this variable, although low statistical power might limit the ability to draw conclusions. Referring to existing knowledge, studies have shown that persons with disability face barriers in accessing sexual and reproductive health services (for a systematic review in sub-Saharan African countries, see Ganle et al., 2020). More specifically in terms of beliefs, Kassa et al. (2016) measured knowledge–attitudes–practice (KAP) related to sexual and reproductive health of young people with disability in Ethiopia. The authors show that the type of disability is significantly associated with the level of awareness; respondents with hearing or visual impairment had higher levels of awareness than respondents with partial mental impairment. Because in our study, we considered only physical disability, it is possible that different results would have been obtained if the definition of disability had included mental impairment. However, other studies only focusing on physical, visual, or hearing impairment showed a particular vulnerability to sexual violence (Burke et al., 2017) and advocate for the need of future research.

2. Most importantly, this study aimed to also offer practical implications in terms of the current belief or attitude structure around beliefs, attitudes, and behaviors within sexual and reproductive health and rights (SRHR) and be able to develop tailored interventions in each village to specific groups of people. The study was mainly done to ensure that interventions were evidence-based—based on current mindsets and understanding of SRHR behaviors as well as strategies to address unhealthy or unsafe behaviors. The team chose SJTs as a means to specifically pinpoint who was supporting harmful behaviors in which village. While we may see a strong support against child marriage in one village, we may see the opposite belief in an adjacent village. The SJTs were a helpful tool for Sierra Leone Red Cross to ascertain where to focus their efforts for each topic, including with which target group within a specific community. It is also possible to use SJTs in order to identify who is supportive of the desired behavior and could act as peers in the program. That is powerful information for development teams by ensuring that precious funding in health promotion is more likely to deliver behavior change and thus impact.

### Implications

Because we assessed norms toward sexual and reproductive health, methods to change social influence, such as resistance to social pressure and mobilizing social support (Table 6, Kok et al., 2016); methods to change social norms, such as mass media role modeling and mobilizing social networks (Table 10, Kok et al., 2016), and methods to change communities, such as community development and social action (Table 13, Kok et al., 2016) are suggested. Intervening not only at the individual level, but also interpersonal, community, and policy levels would increase the chances of success, as individual behaviors are embedded within a wider context (Veer et al., 2020). Goldmann et al. (2019) provide examples of social norms programs on violence against women prevention and HIV at each level of the ecological model (see their Figure 1, p. 54).

Validity of SJTs answers was assessed by examining the correlation with relevant measures included in the survey. Interestingly, there are positive correlations between those different types of measures, but the magnitude of the association is not big, and for some items not significant. This reveals that SJTs and standard self-report items do not measure exactly the same things. Lacking an objective reference value (e.g., observed behavior), it is difficult to determine which type of measure is best. Given the number of limitations of standard self-report measures mentioned in the introduction (Lipnevich et al., 2011; Flaskerud, 2012; Chyung et al., 2018), we suggest more studies should investigate the validity and usefulness of SJTs to assess beliefs and norms in a community. Scheel et al. (2021) argued that more attention dedicated to validate measures would improve studies’ quality.

Situational judgment tests have also the potential to be use for monitoring progress, if used repeatedly. After being used for baseline assessment (as done in the study reported here), the same SJTs could be used again after a social and behavior change intervention. The answers’ evolution (or lack of) would indicate if the intervention reached its goal or not in terms of modifying norms and beliefs. To our knowledge, SJTs have not yet been used for this purpose, and validation is needed (e.g., do people “allow” themselves to answer differently to the same vignette when presented the second time? Are there any bias associated with the use of SJTs for monitoring?).

### Limitations

The present study has some limitations. Notably, despite an important global sample size (566), the number of response for each SJT was much lower due to skip logic being introduced to the surveys to reduce their overall length for each respondent. This lack of respondents become particularly problematic when testing for interactions with respondents characteristics, as the number of participants in each category dropped sometimes below 10. Thus, future studies need to take into account this sub-division of respondents to ensure a minimum number of responses to perform the analysis. In addition, the fact that some questions were asked only to men or only to women do not allow to compare both groups’ norms on the same hypothetical scenario. Future studies could consider asking the same questions to both groups by asking, if the scenario presents a male protagonist, what female respondents think the men in the scenario should do, and vice versa if the protagonist is a female.

Another limitation is the decision to pre-develop the initial SJT tool for the Sierra Leone local team. The rationale was around capacity—in terms of time and knowledge at the field level to develop behavior change monitoring and evaluation tools. The team member presenting the tool had limited time in-country to train enumerator and health team members or to train those teams to support development of SJTs given limited baseline knowledge of developing protocols in behavior change. The team agreed that an initial tool would be developed for the local team to receive training about, and then led in discussions with the guidance and facilitation to edit as needed to ensure that the tool truly reflected the local contexts. It is important to note that when asked to reflect on the SJTs, the participants all felt that the scenarios were realistic to their settings and represented real dilemmas.

Another limitation is that all SJTs were translated in real time by enumerators. The survey was written in English and in the enumerator training practiced in Krio. The Sierra Leone Team disagreed that the survey should be translated into Krio because they were also, when needed, translating the SJTs and other questions into other local languages (including Mende and Kono). This likely affects the standardization of the SJTs being asked, especially with enumerators being asked to explain options for clarity.

## Conclusion

To conclude, this paper provides pioneer data on norms around sexual and reproductive health issues measured with situational judgment tests, offering a new perspective on those themes and a more targeted approach to assessing and responding to existing beliefs, attitudes, and behaviors around SRHR. FGM is the domain for which the norms seem the most problematic, and age is the factor the most important to consider for tailoring when building social and behavior change interventions.

## Consortium Sierra Leone Red Cross Society Members

Kono Branch: Tamba Palallay – Branch manager; Komba Morsay – Field Health Officer; Amara Keita – Coach; Hannah Boima – Volunteer; Saffiatu Sumana – Volunteer; Kai Philip Morsay – Volunteer; Magnus Lahai – Supervisor; Moyamba Branch: Mohamed Lamin - Branch manager; Sylvester Momodu- Field Health Officer; David Johardy – Volunteer; David Kallon- Volunteer; Massah Pessima – Volunteer; Momodu Mansaray- Volunteer; Rubiatu Nicol – Supervisor; Kenema Branch: Jonathan Kangoma – Branch Manager; Aminata B Musa – Field Health Officer; Thomas Baimba – Coach; Veronica Tengbeh- Coach; Salamatu Vandy – Coach; Sheku Kendebo – Volunteer; Stella Tucker – Supervisor; Bo Branch: Augustine Ellie- Branch Manager; Edward Kemokai – Field Health Officer; Mustapha k Turay – Volunteer; Baindu Sesay – Volunteer; Hassan K Turay – Volunteer; Michael Saidu – Volunteer; Nelson Nyadamoh – Supervisor; Pujehun Branch: Christian Kaipindi – Field Health Officer; Magnus Tiffa – Coach; Mohamed S Kamara – Coach; Abass Koi Fawundu- Coach; Abass Fowai – Volunteer; Momoh Koroma – Volunteer; Samuel Parker – Supervisor.

## Data Availability Statement

The raw data supporting the conclusions of this article will be made available by the authors, without undue reservation.

## Ethics Statement

Ethical review and approval was not required for the study on human participants in accordance with the local legislation and institutional requirements. The participants provided their informed consent to participate in this study.

## Author Contributions

NC, EL, and HV: conceptualization. LM: formal analysis and visualization. SI, AS, and Consortium: investigation. NC: methodology. Consortium: resources. OD and HV: supervision. LM (lead), NC (supporting), and EL (supporting): writing original draft. NC, EL, HV, OD, and LM: writing–review and editing. All authors contributed to the article and approved the submitted version.

## Funding

Ministry of Foreign Affairs of Finland funded data collection. Finnish Red Cross and Sierra Leone Red Cross funded technical coordination. Open access funding provided by University of Geneva.

## Conflict of Interest

NC was employed by company Nadulpan.

The remaining authors declare that the research was conducted in the absence of any commercial or financial relationships that could be construed as a potential conflict of interest.

## Publisher’s Note

All claims expressed in this article are solely those of the authors and do not necessarily represent those of their affiliated organizations, or those of the publisher, the editors and the reviewers. Any product that may be evaluated in this article, or claim that may be made by its manufacturer, is not guaranteed or endorsed by the publisher.

## Footnotes

^1^This study is part of a larger research project. The survey contained items relative to nutrition, water and hygiene, mosquito net usage, and disasters preparedness, and climate change adaptation.
