# Supplementary material for: Sexual and Reproductive Health: How Can Situational Judgment Tests Help Assess the Norm and Identify Target Groups? A Field Study in Sierra Leone
Source: Front Psychol. 2022 May 5;13:866551. doi: 10.3389/fpsyg.2022.866551 (PMC9119186; doi:10.3389/fpsyg.2022.866551)
Supplement: Supplementary file 1 [file Table_1.DOCX]

Supplementary Material

# Appendix A

SJT’s development process:

One of the authors worked with anchoring vignettes and SJTs previously in a project on identifying attitudes and norms around the three key areas for soft skills in youth. The three areas where key soft skills were identified as critical areas for support fell into three categories: Workforce success, violence prevention, and sexual and reproductive health (see Figure S1). The three key areas for youth development correlated closely with many of the issues and behaviors under study by the Red Cross team in Sierra Leone.


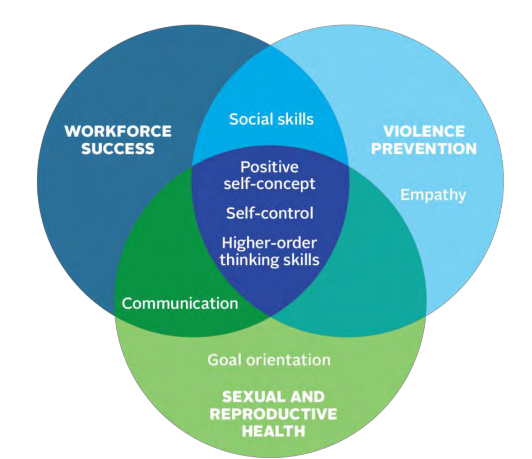


**Supplementary Figure 1.** Critical areas of development for youth (Galloway et al. 2017)

In the youth project, SJTs were developed to present respondents with a scenario intended to test their belief / mindset / judgement related to a specific skill or issue and asking them to respond. They were encouraged to respond to a series of choices they were given about the situation. The SJT tool is developed by prompting people from the target audience to describe specific ‘critical incidents’ pertaining to the skill or issue under study and different potential responses. Often one response is seen as the ‘best’ choice from a social and behavioral perspective, or at least in alignment with the health behavior being advocated. The other possible responses may not necessarily rank in a specific best-to-worst order.

Another option the team considered was the use of anchoring vignettes which present hypothetical situations and people that illustrate different skill levels around an issue, followed by a series of responses. Additionally, at the same time that they are responding to the questions, they are asked to also complete a self-report of their own personal practices related to each issue posed. Respondents would be asked to rank the vignette options provided in order from best to worst and then the assessments are compared to the respondent’s self-report.

These two methods were discussed as an innovative way to measure behavioral beliefs and norms. When the discussion of interviewing people at community level about sensitive and stigma-riddled behaviors within gender-based violence and sexual and reproductive health and rights, several of the authors convened to discuss best strategy. A number of different tools and strategies were proposed including the use of anchoring vignettes, however, the consideration of asking respondents to complete a self-report around the sensitive topics that we aimed to analyse did not seem feasible for the populations we would be interviewing. The team then discussed the development of a SJT with some modification to incorporate the scoring mechanism of ‘best’ answers to the SJT responses to allow quantification of the existing and the desired behaviours. While none of the team in this initial discussion was from Sierra Leone, all were familiar with the Sierra Leone context, having worked there previously on these very topics in previous fieldwork. We agreed to develop an initial SJT tool to share with the Sierra Leone team via one of the team members who would be spending three weeks with the Sierra Leone team in one months’ time. When the team member arrived in Sierra Leone, she would train the team on key social and behavior change topics, tools and health promotion around the key areas under discussion, namely child/early/forced marriage (CEFM), gender-based violence (GBV), family planning, and female genital mutilation (FGM). The Sierra Leone team would then work together with the visiting team member to discuss the proposed SJT tool, discuss what it was meant to measure and how the data it yielded would help to inform their project behavior change strategy within health programming activities.

References for Appendix Section:

Galloway, T, Lippman, L., Burke, H., Diener, O., and Gates, S. (2017). Measuring Soft Skills & Life Skills in International Youth Development Programs: A Review and Inventory of Tools. Washington, DC: USAID’s Youth Power Implementation IDIQ- Task Order 1, Youth Power Action.
